# Authorities Consulted

**Published:** 1884

**Authors:** 


### AUTHORITIES CONSULTED.

1. Allen's Encyclopædia of Pure Materia Medica.
2. Bönninghausen's Whooping Cough.
3. Bönninghausen's Intermittent Fever.
4. Bönninghausen's Therapeutic Pocket-Book.
5. Simmon's Cough Repertory.
6. Jahr's Symptomen Codex.
7. Hering's Condensed Materia Medica.
8. Hering's Analytical Therapeutics.
9. Gross' Comparative Materia Medica.
10. Lippe's Text-book of Materia Medica (and private communications).
11. Jahr and Possart's Repertory.
12. C. Lippe's Repertory.
13. Hahnemann's Materia Medica Pura.
14. H. N. Guernsey's Lectures on Materia Medica, and Notes on Cough Characteristics, *Hahnemannian Monthly*, vol. viii, p. 322.
15. H. V. Miller, *Hahnemannian Monthly*, vol. viii, p. 309.
16. Hull's Jahr. Repertory and Symtomatology.
17. Raue's Annual Record, six volumes.
18. N. A. J. of Homœopathy, twenty-eight volumes.
19. R. R. Gregg's Illustrated Repertory.
20. The Cypher Repertory.
21. Hart's Repertory of New Remedies.
22. Bell and Laird on Diarrhœa.
23. P. P. Wells on Typhoid Fever.
24. Bryant's Pocket Repertory.
25. Dunham's Lectures.
26. The Organon, three volumes.
27. Cowperthwaite's Materia Medica.
28. Teste's Materia Medica.

